# Randomised trial of neonatal hypoglycaemia prevention with oral dextrose gel (hPOD): study protocol

**DOI:** 10.1186/s12887-015-0440-6

**Published:** 2015-09-16

**Authors:** Jane E Harding, Joanne E Hegarty, Caroline A Crowther, Richard Edlin, Greg Gamble, Jane M Alsweiler

**Affiliations:** Liggins Institute, University of Auckland, Auckland, New Zealand; Newborn Services, Auckland City Hospital, Auckland, New Zealand; Department of Paediatrics: Child and Youth Health, University of Auckland, Auckland, New Zealand

**Keywords:** Hypoglycaemia, Oral dextrose gel, Neonate, Randomised controlled trial

## Abstract

**Background:**

Neonatal hypoglycaemia is common, affecting up to 15 % of newborn babies and 50 % of those with risk factors (preterm, infant of a diabetic, high or low birthweight). Hypoglycaemia can cause brain damage and death, and babies born at risk have an increased risk of developmental delay in later life.

Treatment of hypoglycaemia usually involves additional feeding, often with infant formula, and admission to Neonatal Intensive Care for intravenous dextrose. This can be costly and inhibit the establishment of breast feeding.

Prevention of neonatal hypoglycaemia would be desirable, but there are currently no strategies, beyond early feeding, for prevention of neonatal hypoglycaemia. Buccal dextrose gel is safe and effective in *treatment* of hypoglycaemia. The aim of this trial is to determine whether 40 % dextrose gel given to babies at risk prevents neonatal hypoglycaemia and hence reduces admission to Neonatal Intensive Care.

**Methods/design:**

Design: Randomised, multicentre, placebo controlled trial.

Inclusion criteria: Babies at risk of hypoglycaemia (preterm, infant of a diabetic, small or large), less than 1 h old, with no apparent indication for Neonatal Intensive Care Unit admission and mother intends to breastfeed.

Trial entry & randomisation: Eligible babies of consenting parents will be allocated by online randomisation to the dextrose gel group or placebo group, using a study number and corresponding trial intervention pack.

Study groups: Babies will receive a single dose of 0.5 ml/kg study gel at 1 h after birth; either 40 % dextrose gel (200 mg/kg) or 2 % hydroxymethylcellulose placebo. Gel will be massaged into the buccal mucosal and followed by a breast feed.

Primary study outcome: Admission to Neonatal Intensive Care.

Sample size: 2,129 babies are required to detect a decrease in admission to Neonatal Intensive Care from 10–6 % (two-sided alpha 0.05, 90 % power, 5 % drop-out rate).

**Discussion:**

This study will investigate whether admission to Neonatal Intensive Care can be prevented by prophylactic oral dextrose gel; a simple, cheap and painless intervention that requires no special expertise or equipment and hence is applicable in almost any birth setting.

**Trial registration:**

Australian New Zealand Clinical Trials Registry - ACTRN 12614001263684.

## Background

### Significance of the project

Neonatal hypoglycaemia is common in the first few days after birth. Up to 15 % of normal newborn babies will have low blood glucose concentrations [1]. However, the incidence in babies who have risk factors is much greater: up to 50 % in infants of diabetic mothers, [2] large and small babies [3] and 66 % in preterm babies [4].

Glucose is the primary energy source for the brain, and neonatal hypoglycaemia is associated with brain damage and death [4–6]. Babies born at risk for neonatal hypoglycaemia have an increased risk of developmental delay in later life [7–10]. Indeed, it has been reported that neonatal hypoglycaemia is the only neonatal morbidity independently associated with later developmental delay in late preterm babies [11]. While it is uncertain what degree or duration of hypoglycaemia is necessary before morbidity occurs, it is known that even babies without symptoms can have adverse outcomes [4, 6]. Thus hypoglycaemia is common and the only readily preventable cause of brain damage in the newborn.

### Standard management

Blood glucose concentrations normally fall in the first 1–2 h after birth, and then begin to rise again as babies mobilise their body stores of fat and glycogen and begin to feed. In some babies, this physiological fall in blood glucose concentration may persist and, if untreated, potentially may cause permanent brain damage. Since hypoglycaemia is often asymptomatic, the recommended approach is to monitor blood glucose concentrations in all babies at risk, usually by repeated heel-prick blood samples, commonly 4 hourly, in the first 1–2 days [12, 13]. This is painful for the baby and distressing for all concerned.

It is generally accepted that blood glucose concentrations < 2.6 mmol/L require treatment [4, 14, 15]. Standard management of babies in whom low glucose concentrations are detected is to minimise the duration of hypoglycaemia and ensure the glucose is ‘normalised’ as quickly as possible [6, 16]. This commonly requires admission to Neonatal Intensive Care Unit (NICU) for intravenous glucose, separating mother and baby and delaying the establishment of breast feeding as well as incurring high healthcare costs.

The American Academy of Pediatrics advises early identification of the at-risk baby and institution of prophylactic measures to prevent neonatal hypoglycaemia [12]. This is commonly achieved by early feeding, often with supplemental formula milk [12, 17]. However, supplementing with formula milk has been shown to reduce longer term breastfeeding rates [18]. Furthermore, there are both human and experimental data indicating that supplementation of newborns in the first two weeks may have long-term effects on metabolic outcomes. Even brief periods of nutritional supplementation in preterm babies result in altered control of blood pressure and insulin regulation in adolescence [19, 20]. Thus, interventions that prevent hypoglycaemia without supplemental, artificial feeds may help maintain breastfeeding and also have benefits for both neurodevelopmental and metabolic outcomes.

### Recent advances

#### Oral dextrose gel

Harris et al. demonstrated that treatment of neonatal hypoglycaemia with oral dextrose gel was more effective than feeding alone in reversing the hypoglycaemia, and also reduced the rate of NICU admission for this problem and reduced the rate of formula feeding at two weeks of age [21]. Importantly, the gel was well-tolerated, cheap, simple and safe to administer, and was acceptable to families and caregivers.

### Aim

We therefore propose a randomised controlled trial to determine if prophylactic oral dextrose gel given to newborns at risk prevents neonatal hypoglycaemia and thus reduces NICU admission, improves breast feeding rates and reduces costs as well as potentially reducing the risk of later adverse outcomes.

### Hypothesis

The primary hypothesis of this study is that, compared to placebo and standard care, prophylactic 40 % oral dextrose gel given to babies at risk of hypoglycaemia (infant of diabetic mother, preterm, large or small for dates, or other) reduces admission to the Neonatal Intensive Care Unit.

## Methods/design

### Ethics

Ethics approval has been obtained from the Health and Disability Ethics Committees of New Zealand (ethics reference 13/NTA/8) and by the local institutional research review committees for each centre. The ethics committee is notified of any amendments to the study protocol.

### Study design

A multicentre, randomised, placebo controlled trial comparing 40 % dextrose gel with placebo to prevent hypoglycaemia in the first 48 h in babies born at risk.

## Study population

### Inclusion criteria

Babies born at risk of hypoglycaemia, defined as satisfying at least ONE of the following:

1. Infants of diabetic mothers (any type of diabetes)
2. Preterm (< 37 weeks’ gestation)
3. Small (< 2.5 kg or < 10th centile on population or customised birthweight chart)
4. Large (> 4.5 kg or > 90th centile on population or customised birthweight chart)

AND satisfy ALL of the following:

1. ≥ 35 weeks’ gestation
2. Birth-weight ≥ 2.2 kg
3. < 1 h old
4. No apparent indication for NICU admission at time of randomisation
5. Unlikely to require admission to NICU for any other reasons e.g. respiratory distress
6. Mother intending to breast-feed

### Exclusion criteria

1. Major congenital abnormality
2. Previous formula feed or intravenous fluids
3. Previous diagnosis of hypoglycaemia
4. Admitted to NICU
5. Imminent admission to NICU.

### Primary outcome

#### Admission to NICU

This is defined as admission to NICU (or Special Care Baby Unit (SCBU) for the hospitals which use that name) for > 4 h [22].

### Secondary outcomes

1. Hypoglycaemia (any blood glucose concentration < 2.6 mmol/L in the first 48 h);
2. Admission to NICU for hypoglycaemia;
3. Hyperglycaemia (any blood glucose concentration > 10 mmol/L);
4. Breastfeeding at discharge from hospital (full or exclusive);
5. Received any formula prior to discharge from hospital;
6. Formula feeding at 6 weeks of age;
7. Cost of care until primary discharge home;
8. Maternal satisfaction (via telephone questionnaire at 6 weeks);
9. Neurosensory disability at 2 years’ corrected age (any of: legal blindness; sensorineural deafness requiring hearing aids; cerebral palsy; Bayley Scale of Infant Development Version III cognitive, language or motor score lower than one standard deviation below the mean).

## Trial entry

### Informed consent

Parents of babies who are likely to become eligible (maternal diabetes, likely late preterm birth, or anticipated high or low birth weight) will be identified through lead maternity carers and antenatal clinics and provided with an information sheet as early as is feasible. Written informed consent will normally be obtained before the birth.

## Randomisation

Eligible babies for whom consent has been obtained will be enrolled and randomised immediately after birth. Babies will be assigned randomly via an internet randomisation service to the dextrose or placebo group with priority stratification for collaborating centre and risk factor (i.e. maternal diabetes, preterm, small or large).

### Discontinuation of randomised treatment

The allocated treatment can be stopped at any time at the request of the parents, or by the neonatologist caring for the baby if (s) he feels that stopping the treatment would be in the best interest of the baby. The baby will still be followed up and analysed according to the intention-to-treat principle.

## Study groups

The study intervention drug (both dextrose gel and placebo) will be supplied by Biomed Ltd (Auckland, New Zealand) in identically labelled, pre-filled syringes of either 40 % dextrose gel or identical appearing 2 % hydroxymethylcellulose placebo gel in individually pre-labelled trial packs. Each participating centre will have a supply of study packs held in a medications fridge. The staff member randomising the baby will receive a study number corresponding to a pre-labelled study pack, and also the volume of gel (0.5 ml/kg) to be administered. The inside of the baby’s cheek will be dried with a gauze swab, and the study gel massaged into the buccal mucosa at one hour after birth.

In order to determine the most effective dose in prevention of neonatal hypoglycaemia, we undertook a dosage trial (registered with the Australian New Zealand Clinical Trials Registry ACTRN12613000322730). The dose selected of 0.5 ml/kg (200 mg/kg) 40 % dextrose gel at 1 h of age had maximal efficacy in prevention of hypoglycaemia, with the fewest limitations (i.e. was easy to administer, well tolerated and with minimal additional workload or financial cost).

### Blood glucose analysis

The initial blood glucose concentration will be measured at 2 h, as is common practice. Subsequent management will be according to hospital standard practices. All blood glucose concentrations will be analysed by the gold standard glucose oxidase method, either with a portable blood glucose analyser (e.g. iSTAT, Abbott Laboratories, Abbott Park, IL USA) or a combined metabolite/blood gas analyser (e.g. ABL 700, Radiometer Ltd, Copenhagen, Denmark).

## Follow up after birth until time of discharge from hospital

### Surveillance

Babies will be monitored according to routine clinical practice. This includes pre-feed blood glucose measurements 2–4 hourly for at least the first 12 h, and until there have been 3 consecutive measurements > 2.6 mmol/L. If the baby becomes hypoglycaemic (blood glucose concentration < 2.6 mmol/L) then the hospital protocol will be followed for management of hypoglycaemia, including the administration of treatment dextrose gel and supplementary feeds if relevant. We will monitor for serious adverse events (seizures and death) and other adverse events; hyperglycaemia (as above), late hypoglycaemia (blood glucose concentration < 2.6 mmol/L for the first time after 12 h of age), delayed feeding (failure to establish breastfeeding without supplements by the end of day 3) and systemic sepsis [22].

## Follow-up after primary hospitalisation

Parent (s) of participants will be contacted on day 3 (if already discharged home) and 6 weeks after birth to complete a telephone questionnaire. This will include details of the current feeding regime, and at 6 weeks, parental satisfaction with participation in the trial and health status of the baby. We will maintain contact with babies and their families. At 2 years’ corrected age all families will be contacted to arrange a developmental assessment to investigate longer-term outcomes. We will provide results when available to those who have informed us that they wish to be made aware of the outcome of the trial.

## Data analysis

The primary outcome of NICU admission will be analysed by logistic regression, stratifying by collaborating centre. Secondary analyses will adjust for potentially confounding variables: reason for risk of hypoglycaemia (infant of diabetic, late preterm, small or large), sex, gestational age, and mode of birth (vaginal vs caesarean section). Continuous data will be compared by Student’s *t* test, or the Mann–Whitney *U* test if the data are not normally distributed and cannot be converted to near-normality by simple transformation. Data with repeated points, such as blood glucose concentrations, will be compared using mixed model techniques, modelling the main effect of treatment group allocation, time and their interaction, with significant main effects and interactions tested using the method of Tukey. All tests will be two-tailed, with P < 0.05 considered significant. The data will be analysed on an intention-to-treat basis, and any baby who dies or for whom the primary outcome of NICU admission cannot be determined will be assigned the worst case outcome of NICU admission.

### Economic evaluation

The cost-effectiveness of oral dextrose gel to prevent neonatal hypoglycaemia will be compared with usual care (no prophylaxis) within the period to discharge.

Intervention costs: A per-baby cost of dextrose gel will be based on the cost of the gel syringes and dispensing costs. For the no prophylaxis option, a zero intervention cost will be assumed.

Other Hospital Costs: Resource utilisation will be obtained from a clinical record form identifying both length of stay (LOS) and relevant Diagnostic Related Group (DRG) code for the mother, plus any subsequent operative procedure (DRG), respiratory problem requiring treatment (DRG), and NICU admission for the baby (plus LOS). Costs will be assessed using New Zealand Ministry of Health cost weights and purchase unit prices. For the no prophylaxis option, the costs from the placebo gel arm will be used.

Cost-effectiveness will be assessed using incremental cost-effectiveness ratios (ICERs) formed in terms of an incremental cost per case of hypoglycaemia avoided. Uncertainty in these figures will be assessed using non-parametric bootstrapping (sampling with replacement) to form a distribution for the ICER, potentially including corrections for any differences in the composition of the trial arms in any confounding factors. This analysis will be presented using cost-effectiveness acceptability curves that identify the likelihood of each option being cost-effective for different values attached to reducing a case of hypoglycaemia.

### Power and sample size

Based on data from Auckland City Hospital and Waikato Hospital, 10 % of at-risk babies will require admission to NICU. A trial of 2,129 babies (1,014 in each arm, with continuity correction and allowing for 5 % drop-out rate), will have 90 % power to detect a 40 % relative reduction (absolute reduction of 4 %) in admission to NICU from 10–6 % with two-sided alpha of 0.05.

Should this study show that dextrose gel is effective at preventing NICU admission, it will be critical to find out if this also improves long-term outcomes for babies. A sample size of 2,014 babies would allow us to detect a reduction in the number of children with one or more low developmental scores (<1SD below the mean) on a Bayley Infant Scales of Development Edition III assessment at 2 years from 40–33 %.

## Discussion

This study is the first to investigate whether neonatal hypoglycaemia and admission to NICU can be prevented by oral dextrose gel, a simple, cheap and painless intervention. This intervention requires no special expertise or equipment and hence is applicable in almost any birth setting. Reduction of admission to NICU will reduce separation of mother and baby, and may improve breastfeeding rates and reduce financial cost.

## Abbreviations

CI: Confidence interval
DRG: Diagnostic related group
hPOD: Hypoglycaemia Prevention in newborns with Oral dextrose
ICER: Incremental cost-effectiveness ratio
LOS: Length of stay
NICU: Neonatal intensive care unit
NHI: National health identifier
P: *P*-value
Pre-hPOD: Pre-hypoglycaemia Prevention in newborns with Oral Dextrose/dosage trial
SCBU: Special care baby unit
SD: Standard deviation
